# Comparing Zirconium Crown Marginal Adaptation in Preparations with Two Different Occlusal Reductions

**DOI:** 10.3390/dj12030077

**Published:** 2024-03-19

**Authors:** Ali Khekan, Bernd Kordaß

**Affiliations:** Department of Prosthodontics, Faculty of Dentistry, Greifswald University, 17489 Greifswald, Germany; kordass@uni-greifswald.de

**Keywords:** marginal fitness, zirconia, occlusal reduction scheme, resin-modified glass ionomer cement

## Abstract

This study aimed to assess and contrast the effects on the vertical marginal fit of full contour CAD/CAM-generated monolithic zirconia crowns at pre- and post-cementation levels with various occlusal reduction schemes (planar and flat) and cements. Forty sound human maxillary first premolars were sampled for this study. The samples were divided into two main groups with twenty samples in each group according to the occlusal reduction scheme as follows: Group A included a chamfer finishing line design with a planar occlusal reduction scheme and Group B included a chamfer finishing line design with a flat occlusal reduction scheme. Each group was sampled into two subgroups (*n* = 10) based on the type of cement as follows: resin-modified glass ionomer cement (Fuji Plus) for subgroups A1 and B1, and a universal adhesive system (Duo Estecem II) for subgroups A2 and B2. Marginal gaps were tested in four indentations using a Dino light stereomicroscope (230×). Paired *T*-tests and Student’s *t*-tests were used to analyze the data. Before cementation, subgroup A1 scored the lowest mean of vertical marginal gap values, while subgroup B2 scored the highest mean; following cementation, subgroup A1 scored the lowest mean of vertical marginal gap values, and subgroup B2 scored the highest mean of vertical marginal gap values. A chamfer finishing line design with a planar occlusal reduction scheme could be a preferable occlusal reduction scheme.

## 1. Introduction

The marginal fitness of crown restoration is one of the crucial factors for the success of prosthetic restoration, along with the clinical quality, longevity, and predictability of a dental prosthesis. Accurate assessment and quantification of marginal parameters are needed for their measurement to distinguish between fit and misfit [1]. Poor marginal fit of the restoration can cause harm to the tooth, periodontal tissue, and even the restoration itself. A significant marginal discrepancy can cause cement to dissolve, microleakage to occur, and plaque to build up, which causes pulpal lesions, caries, and gingival inflammation [2,3].

A marginal gap (MG) is a vertical measurement from the restoration margin to the outermost edge of the finish line of the tooth margin. The absolute marginal discrepancy (AMD) refers to the distance between the crown’s margins and the preparation’s Cavo surface angle, or the angular combination of the vertical and horizontal marginal discrepancies [4]. Different studies have recommended a marginal space between 50 μm and 120 μm as acceptable, while other studies have recommended a spacing of less than 100 μm [5,6]. The authors found that restorations with an MD of less than 120 μm had a higher success rate in in vivo research involving more than 1000 crowns [7].

The reported MD for crowns made using CAD/CAM technology ranges from 50 to 100 μm [8,9]. Dental professionals were able to employ new treatment modalities and alter the design and application parameters of all-ceramic restorations with the advent of computer-aided design and computer-aided manufacturing (CAD/CAM) technology, which coincided with an increase in posterior oral aesthetic demand [10]. Clinical evidence supports the use of CAD/CAM machining for dental restorations. When compared with the use of impression materials, the digital system has many advantages over the traditional method, such as the possibility of digital articulation, reduced cost, procedural complexity, and waste, enhanced patient acceptance, and the removal of the necessity for manual pouring with cast trimming and impression disinfection [11]. A single-use, disposable camera sleeve is only needed for infection control. The digital system also does away with the need for trays for impression, delivers digital files electronically, and eliminates the need for physical shipping, which would subject a physical impression to humidity fluctuations, temperature, and time. The digital impression is then ready for design without the anticipated dimensional changes in the conventional system [12]. For fixed partial dentures (FPDs) and crowns, it is a common belief amongst dental professionals that some types of teeth require rehabilitation more frequently than others. However, there is no actual study on the different types of teeth involved.

Numerous advancements in ceramic restoration have resulted from growing demands for great aesthetics and biocompatibility [13]. The German scientist Martin Heinrich Klaproth first recognized zirconia in 1789. Then, in 1824, a Swedish chemist named Jons Jakob Berzelius was the first to create impure zirconium metal by boiling potassium and potassium fluoride [14]. High-strength zirconia is applied to produce fixed partial prostheses, even in load-bearing areas. Materials based on zirconia offer several benefits, including ideal mechanical qualities, limited bacterial adherence, conventional cementation, and biocompatibility. However, mechanical characteristics are not the sole factor that contributes to dental restorations’ excellent endurance. It is possible to classify zirconia CAD/CAM crown restoration according to the type of fabrication of partial contour zirconia. Veneered zirconia crowns are made using partially sintered Y-TZP blocks. Researchers have demonstrated that monolithic zirconia crowns resist posterior masticatory stresses and do not break the veneering ceramic as their veneering counterparts did. During the virtual three-dimensional (3D) design of the repairing part, 22–24 CAD-CAM system settings enable the adjustment of various factors, such as cement spacing and the thickness of the restorative material. Additionally, shaded monolithic zirconia blocks help in the creation of restorations with better translucency thanks to newly developed technologies and altered production techniques [15].

A partial-contour zirconia restoration involves milling a durable zirconia framework and covering it in a more aesthetically pleasing porcelain veneer [16]. Also, for full-contour zirconia: partially sintered Y-TZP blocks create full-contour anatomical crowns. One material block can mill a monolithic unit for full-contour restorations without porcelain overlays [17,18]. It has been used with crowns over implants, posterior crowns, full-arch bridges up to 14 units, and crowns with limited occlusal clearance. It is also advised that any anterior restoration made of zirconia employ a porcelain face veneer for aesthetic purposes. It has various characteristics, including flexural strength and fracture toughness, low thermal expansion numbers, resistance to thermal shock, better esthetics, and wear compatibility [19]. Full-contour zirconia, on the other hand, can be utilized in anterior situations when the dentist wants to prioritize the restoration’s strength over its aesthetics. It has numerous qualities including enhanced aesthetics, wear compatibility, resistance to thermal shock (low thermal expansion numbers), high flexural strength, and fracture toughness. Even if the material used for an indirect restoration plays a crucial role in its performance, it is useless without high-quality cement and the correct cementing technique to prevent oral fluids from getting into the space between the restoration and the tooth.

Marginal fit accuracy could be affected by different elements, such as variations in CAD-CAM production lines concerning scanning accuracy, CAD software performance, zirconia condition during milling, or the CAD-CAM system grinding procedure. Furthermore, hand changes made by dental technicians to CAD-CAM restorations enhance the restoration fit after finishing the milling, as we explained before [20].

In conclusion, after the mechanical and thermal aging of monolithic zirconia crowns, they can endure much higher fracture loads than the average maximal occlusal forces [21]. The surface finishing state of monolithic zirconia crowns does not affect their fracture resistance, which is significantly higher than conventional metal–ceramic crowns, even after prolonged artificial aging conditions [22].

Types of zirconia

1. Zirconia-Toughened Alumina (ZTA):

Particles of zirconia can be combined with a matrix of alumina (Al_2_O_3_). These materials make use of the stress-induced changeability of scattered zirconia. In contrast to the other two classes, the stability of the tetragonal phase at room temperature is controlled by the size, shape, and location of the particles (intra- or intergranular) rather than using dopants [23].

2. Magnesia Partially Stabilized Zirconia (Mg-PSZ):

The array of cubic zirconia in the microstructure of Mg-PSZ stabilizes by 8 to 10% mol of magnesium oxide. Magnesium silicates can form with a low magnesia content due to the difficulties in obtaining free silica Mg-PSZ precursors (SiO_2_), which favors the transformation from tetragonal to monoclinic and lowers the material’s mechanical characteristics and stability. This material has been fully sintered into blocks, which require stiff and powerful machining tools. However, these types of zirconia have not been successful due to their porosity and high particle sizes (30–60 μm), which encourage surface wear [24].

3. Yttria Fully Stabilized Tetragonal Zirconia Polycrystal (3Y-TZP):

Due to its outstanding mechanical characteristics, biocompatibility, and aesthetic possibilities, zirconia ceramic is the most frequently used material in dentistry. When used in dental applications, it is made of microstructures with small grains (0.2 to 0.5 μm in diameter), which prevents structural degradation or destabilization in the presence of saliva and slows the development of subcritical cracks [25]. Such restoration can be accomplished by entirely machining the sintered block or milling the pre-sintered block and then sintering at a high temperature. When using a pre-sintered block, the repair is pre-shaped into a size that is 25–30% larger than the intended shape to account for shrinkage during sintering at temperatures between 1350 and 1550 °C [16].

Zirconia CAD/CAM crown restoration according to the type of fabrication.

- Partial-contour zirconia: Veneered zirconia crowns were created using partially sintered Y-TZP blocks. A partial-contour zirconia restoration involves milling a durable zirconia framework and covering it in a more aesthetically pleasing porcelain veneer. These restorations have various names, including veneered, zirconia-based, and bi-layered. Another option is to apply the veneering layer where it is cosmetically necessary; these restorations are known as hybrid or minimally veneered restorations [25].
- Full-contour zirconia: Partially sintered Y-TZP blocks were used to create full-contour anatomical crowns. One material block can mill a monolithic unit for full-contour restorations without porcelain overlays [17,18]. Similar to regular porcelain fused to metal (PFM) crowns, it can be ready for the application of a knife-edge, chamfer, or shoulder finishing line [26]. It is approved for use with crowns over implants, posterior crowns, full-arch bridges up to 14 units, and crowns with limited occlusal clearance. The major candidates are grinders and bruxers who do not want metal occlusal PFM restorations or cast gold.

Also, it is pointed out that any anterior restoration made of zirconia employs a porcelain face veneer for aesthetic purposes. However, in certain anterior cases, when a dentist wants to reinforce the strength of the restoration rather than its aesthetics, complete contour zirconia may be employed. It has various characteristics, including flexural strength and fracture toughness, low thermal expansion numbers, resistance to thermal shock, better esthetics, and wear compatibility [19]. Dental cements are divided into the following two categories: resin-based and water-based polymerizing cements [27]. Zinc phosphate cement and glass ionomer cement are examples of water-based cement, whereas resin-based cement includes resin composites, resin-modified glass ionomer cement, and adhesive cement [28]. The chemical attachment of water-based cement to tooth structures or restoration materials is minimal or nonexistent [29]. Resin cement is the newest type of cement for indirect restorations; it can bond to the tooth structure and the restoration’s inner surface. These types of cement are utilized for cementation of all types of restoration since they are more complex, non-resorbable in oral fluid, and highly technique-sensitive than conventional cement despite having higher tensile, compressive, and flexural properties than other types of cement [30]. Several studies show that the use of adhesive resin composite cement promotes a marginal fit and minimizes micro-leakage [31].

Adhesive agents are commonly used to join ceramic crowns to the prepared stiff tissue foundation to increase retention, marginal adaptation, and fracture resistance of the restored tooth [26]. Based on the available scientific evidence, no consensus exists on the maximum clinically acceptable marginal discrepancy (MD), with reported values varying between 50 and 200 µm. Increased MD values reduce the fracture resistance of the crown and the veneering porcelain [32]. Digital impressions, one of the CAD/CAM technologies, offer speed, accuracy, and high-quality restorations because they are designed based on the properties of the materials, can store information, and can transfer images between the laboratory and the dental practice [33].

As said before, this study aims to assess the impact of different occlusal reduction schemes combined with different types of cement on the marginal fit of full-contour zirconia crowns. The idea was to test the hypothesis that there would be no discernible variation in the marginal fit of monolithic zirconia crowns made with the same CAD-CAM system software but with various occlusal reduction schemes and types of cement.

## 2. Materials and Methods

Forty human maxillary first premolars of similar size and form were selected. We used G power 3.0.10 (a program written by Franz-Faul, University of Kiel, Kiel, Germany) [34] with a power of study = 85%, an alpha error of probability = 0.05 two-sided, and an effect size of F of 0.4 (Large effect size), with four groups and two measurements. Based on all these conditions, the definite sample size is about 40 samples.

The teeth were removed from patients between 18 and 27 years old to undergo orthodontic therapy. To reduce the number of variables in this study, every tooth needed to have a crown that was measured using a modified digital caliper [35], and every tooth needed to be free of cavities, restorations, cracks, and abnormalities in the enamel [36]. The teeth were split into two major groups at random [37]. The roots of the teeth were embedded in cold acrylic resin (20 mm in height and 10 mm in width) up to 2 mm below the cementoenamel junction to simulate the level of alveolar bone height in a healthy tooth, with the help of a plastic mold to facilitate the preparing procedures [38].

**Figure 1:**
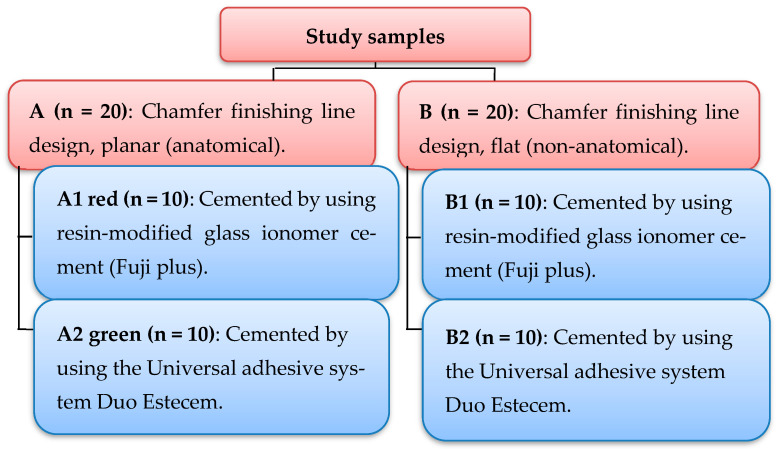
Two primary groups were formed and divided into four subgroups.

Sample grouping: Two primary groups were formed with twenty samples in each group according to the occlusal reduction scheme as follows (Figure 1): 

Group A: chamfer finishing line design with a planar (anatomical) occlusal reduction scheme.

Group B: chamfer finishing line design with a flat (non-anatomical) occlusal reduction scheme.

Next, each primary group was divided into two subgroups based on the type of cement in use.

Subgroup A1 red: crowns cemented using glass ionomer cement (Fuji Plus, Tokyo, Japan).

Subgroup A2 green: crowns cemented using the Universal adhesive system (Duo Estecem II Universal).

Subgroup B1 blue: crowns cemented using glass ionomer cement (Fuji Plus).

Subgroup B2 yellow: crowns cemented using the Universal adhesive system (Duo Estecem II Universal).

For standardization, the same operator prepared each sample with the help of a dental surveyor. The surveyor was tasked with holding the turbine (110.000 round rpm). Preparation for any restoration requires that adequate tooth structure be removed to allow for restoring the tooth to its original contours while ensuring sufficient thickness for the restorative material. Whenever possible, tooth structure should be preserved, but reduction must be adequate to enable the dentist to fabricate a crown of acceptable strength and optimal contours [39]. The bur’s long axis should be perpendicular to the tooth’s long axis and the bur’s convergence with the axial wall of the tooth should be uniform when preparing teeth to receive a zirconia crown following the recommendations for Ivoclar Vivadent, which includes the following preparation features: an axial reduction of 1 to 1.5 mm, a chamfer finishing line of 0.8 to 1.5 mm in depth, an occlusal-gingival height of 4 mm, a convergence angle of 3 o for each axial wall (the total convergence angle of 6 o), and a chamfer height of 0.8 to 1.5 mm above the cementoenamel junction. These dimensions were examined using a digital caliper. Following the axial reduction of each tooth following its group, the occlusal surface was prepared to create a smooth occlusal reduction scheme using a diamond wheel bur (No. 824 047, Komet, Lemgo, Germany). Rugby ball bur (No. 899 314 027, Komet, Lemgo, Germany) was used to further reduce the occlusal surface of all the tooth samples in subgroups A1 and A2 to alter the design into a planar shape. To keep the crown restoration from experiencing stress concentration, all internal line angles and sharp angles were rounded. All fabrication processes, including model scanning, milling, and sintering protocols, were carried out by the Open Techniques desktop scanner (Open Technology, Brescia, Italy). The open scanner is highly customizable and proves to be the best scanning solution for any restoration. The 3D scanner supports must rest on a perfectly flat, horizontal surface (leveling machine).

According to the manufacturer’s instructions, the scanning process started by placing samples inside the 3D scanner by laying them on the chosen model support, which consequently attached to the magnetic joint at the base of the scanner’s opening; the scanning area was placed at a height of 90 mm from the base of the scanner’s opening. The scanning operation then started using management software installed on a computer connected to the 3D scanner. The scan time was 10 s for each tooth.

As the next step, the crown was designed in the “MODEL” phase, which defined the preparation’s boundary that the system automatically detected. Apart from ascertaining the tooth’s placement within the arch and its path along the insertion route, the undercut was also checked. Crown milling parameters were determined in the “DESIGN” phase according to the open Techniques-Desktop scanner as follows:

First, (80 μm) the space (80 μm), (0 μm) offset for occlusal milling.

Then, (500 μm) radially, (700 μm) occlusal, (150 μm) marginal. Finally, all the data were sent to the milling machine. Within the lab (DWX-52D Dental Milling Machine, Hamamatsu, Japan), in the “MILL” phase, dry milling began.

The shaping procedure carried out by the carbide cutting tools functions simultaneously without any interference during the completely automated milling. The milling process for each crown took 10 to 15 min. Once each completed crown was positioned on its matching natural tooth, the restoration’s size and placement within the CAD/CAM blank were determined. The furnace heating temperature and duration were set up in a ZETIN-TECH furnace according to the manufacturer’s specifications. They began at 27 °C, were gradually elevated over the first hour to 1480 °C, remained at that temperature for 30 min, and were then dropped to the starting point once more to achieve their final color, strength, and size. To account for the shrinkage (20–25% greater in size) that happens when firing partially stabilized zirconia, the zirconia crowns were white, chalky, and milled to a larger size [40]. The crowns were placed immediately on the firing stand with their inner surfaces facing up. After the sintering procedure, each crown was cooled to room temperature. Following the sintering process, all crown restorations’ inner surfaces were sandblasted for 15 s following the manufacturer’s specifications and applied at 10 mm, 1 bar for 15 s while utilizing aluminum oxide particles smaller than 50 μm. A rough and retentive surface was required to enhance the mechanical interlocking between the luting cement and zirconia. To hold the specimen on the microscope stage during the measurement of vertical marginal gaps, to fix the crown on the tooth sample, and to maintain the forces during the cementation, a specifically built holding device was designed for this investigation. A load sensor was intended to be affixed to the device. With a typical load of 5 kg, each crown was placed on the tooth sample [3,41]. This load was applied over each sample to replicate the average biting forces produced by the jaw during measurement by simulating a sitting force during crown cementation [42]. The cementation process was based on the manufacturer’s leaflet for both cements. The inner surface of the crown was filled with cement once the cement material had been mixed. Cement was injected into each crown’s intaglio surface using a disposable mixing tip. First, finger pressure was applied to ensure the crown was firmly in place. Subsequently, a vertical static force of 5 kg (about 50 N) was applied to the occlusal surface for 5 min using a specimen-holding apparatus specially made for the purpose. Replication of the biting force during cementation was performed clinically [43]. To reproduce the cushioning effect of the cotton roll used in clinical crown seating and to evenly distribute the load throughout the occlusal surface of the tested crown, a rubber piece was placed between the load applicator and the crown during cementation [44]. The samples were cemented with the Universal adhesive system, utilizing an alight cure unit to spot-cure the cement for 2 s; a probe was used to remove the surplus cement. The sample was then given a 20-s curing treatment on each surface. While the samples were cemented with Fuji Plus glass ionomer cement, approximately 2 min after starting the mixing, the excess was removed as per the manufacturer’s instructions. All specimens were kept in distilled water at room temperature and evaluated 24 h following cementation [45]. Marginal gap measurements were taken at two distinct times as follows: before and after cementing. Measurements were taken by connecting a Dino-Lite digital microscope to a PC with a USB with 230× magnification by Holmes et al. (1989) using the definition of the marginal gap the perpendicular distance from the margin of the finishing line preparation to the margin of the restoration [4].The measurements were taken at four spots on each tooth’s surface (two line’s margins, two points marked with permanent marker in the middle of the surface, and two additional points on the left and right sides, separated by one millimeter from the preceding two points). It was possible to keep the crown–tooth assembly in place using a specimen-holding device that was specifically designed for this purpose. After initially applying steady finger pressure for seating during cementation, the measurements were obtained perpendicular to the tooth axis [46]. The largest gap was selected to reflect the sample’s marginal gap [47]. Every measurement was carried out by the same researcher [48]. The digital microscope was positioned so that its long axis lined up with the long axis of the tooth. The handle was secured, preventing it from being changed vertically without altering the microscope’s horizontal tilt. The image opening in an image-processing application (Image J 1.50i, USA) allowed for the measurement of the marginal gap in pixels after Dino-capture software 50i was used to process two photographs for each surface of the tooth sample. To convert the measurements to micrometers, a digital microscope was used to take a picture of one millimeter of a ruler at a magnification of 230×. After that, the picture was opened in the (Image J) application, and a line that matched a known distance of one millimeter was created using the straight-line selection tool. All estimated measurements were transformed from pixels to µm by opening the Set scale window after selecting the analyze option from the main menu while keeping the microscope’s calibration and magnification intact [49]. The known distance and measurement unit (1000 and µm, respectively) were entered into the dialog box. The distance was automatically entered into the pixels field based on the length of the chosen line [50].

### 2.1. Statistical Analysis

#### 2.1.1. Shapiro–Wilk Statistical Analysis

The statistical analysis was performed using SPSS 21. The following statistical techniques were applied to evaluate and examine the data:

#### 2.1.2. Descriptive Analysis

Minimum, maximum, mean, and standard deviation (SD) for quantitative variables.

A. Statistical tables.

B. Graphical presentation.

#### 2.1.3. Inferential Analysis

1. Student’s *t*-test was used to investigate the causes of the difference and identify any significant differences between the two categories.

2. The marginal gaps for each sample were compared using a paired samples *t*-test.

3. A post hoc/LSD test was performed to identify exactly which groups differ from each other. They are usually used to uncover specific differences between three or more group means when an analysis of variance (ANOVA) test is significant.

The level of significance was considered not significant when *p* > 0.05 and significant when *p* < 0.05.

## 3. Results

The vertical marginal gap was measured from the two main groups (A, B) and their subgroups (A1, A2, B1, and B2) a total of 1280 times, with 16 measurements for each tooth sample (A1, B1, both before and after cementation; A2, B2, before and after cementation). Marginal gap values were recorded in μm for all subgroups’ samples. 

### 3.1. Pre-Cementation Results

Testing the normality of distribution.

The Shapiro–Wilk test was used to determine whether the collected data’s distribution was normal. The data were found to be regularly distributed by the Shapiro–Wilk test (*p* > 0.05) (Table 1).

### 3.2. Descriptive Statistics

A total of 640 marginal gap values were measured in units of μm. The marginal gap’s averages and standard deviations, along with its lowest and greatest values, were determined for every grouping, as shown in Table 2.

**Figure 2:**
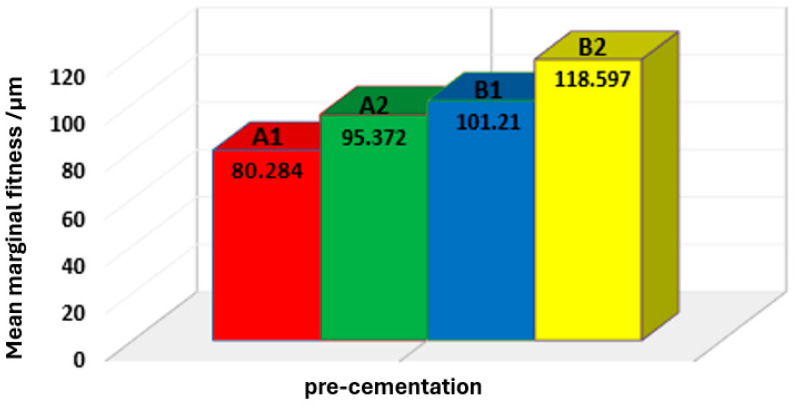
Bar chart showing the mean values of the marginal gap for all subgroups in micrometers.

Table 2 demonstrates that subgroup A1 (80.284 ± 23.021) had the lowest mean of vertical marginal gap values, while subgroup B2 (118.597 ± 9.956) had the highest mean of vertical marginal gap values pre-cementation, and this is clearly shown in Figure 2.

### 3.3. Inferential Statistics

1-Student’s *t*-test was used to see whether there are any statistically significant differences between the four subgroups and to find the significant differences between each pair of subgroups, as shown in Table 3. The current study proved that there are highly significant differences between each of the following subgroups: A1-B2, A2-B1, A2-B2, while there is no significant difference between the remaining three subgroups: A1-A2, A1-B1, B1-B2.

2-Test for least significant difference (LSD) multiple comparisons: According to Table 4, there are significant differences in marginal gaps between all the tested subgroups (A1, A2, B1, and B2). A1 was the lowest one, and B2 was the highest.

### 3.4. Post-Cementation Results

Testing the normality of distribution (Table 5).

Based on the results of this test, all samples that were measured within the groups under study were homogeneous and normally distributed, as the significance value was greater than 0.05 (*p* > 0.05).

### 3.5. Descriptive Statistics

A total of 640 marginal gap values were measured in units of μm. The marginal gap’s averages and standard deviations, along with its lowest and greatest values, were determined for every grouping.

**Figure 3:**
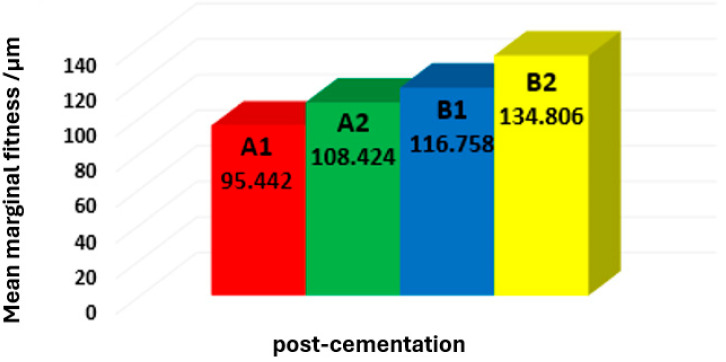
Bar chart showing the mean values of the marginal gap for all subgroups in micrometers.

Table 6 demonstrates that subgroup A1 (chamfer finishing line with planar occlusal reduction scheme) scored the lowest mean of vertical marginal gap values (95.45 ± 17.598), while subgroup B2 (chamfer finishing line with flat occlusal reduction scheme) had the highest mean (134.806 ± 8.114) (Figure 3).

### 3.6. Inferential Statistics

1. Student’s *t*-test was used to detect if there are any statistically significant differences between the four subgroups and to find the significant differences between each pair of subgroups, as shown in Table 7. The current study reported that there are highly significant differences between each of the following subgroups: A1-B1, A1-B2, A2-B2, and B1-B2, while there is no significant difference between the remaining sub-groups: A1-A2, A1-B1, A2-B1.

2. Test for least significant difference (LSD) multiple comparisons: According to Table 8, there is a significant difference in marginal gaps between groups (A1 and A2; B1 and B2), but the difference between A2 and B1 was insignificant. A1 is still the lowest one, and B2 is the highest. It should be noted here that there is no significant difference between the A2 and B1 subgroups.

### 3.7. Comparative Statistical Analysis between Pre- and Post-Cementation

**Figure 4:**
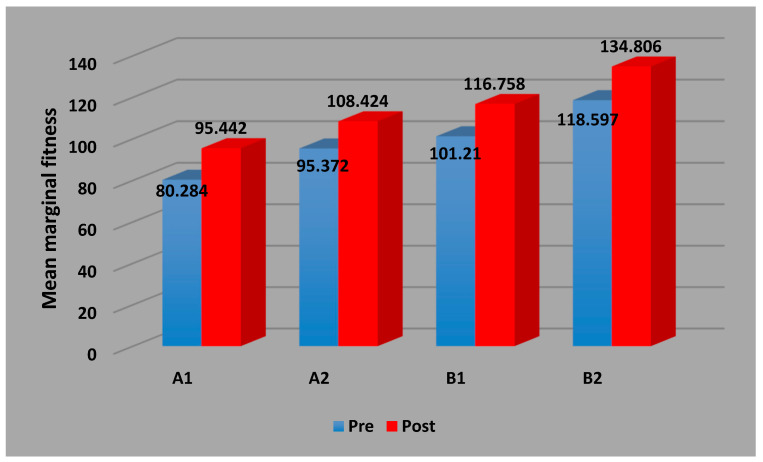
Bar chart showing the mean values of the marginal gap (pre- vs. post-cementation) for all subgroups in micrometers.

1. Student’s *t*-test was performed to investigate the causes of variations and to identify noteworthy distinctions between each pair of subgroups, as shown in Table 9.

2. According to the inferential statistics, there is no significant change in marginal gap in group A1, while the changes are significant in A2, B1, and B2, pre- and post-cementation (Figure 4). 

Marginal fit, when comparing pre-cementation to post-cementation in each group and subgroup, increases when there is a significant change, and the highest increase is in B2, with a slight difference in the other groups.

Among groups (A1-B1, A2-B2), both B1 and B2 have a higher MF than A1 and A2, with a significant difference in both pre- and post-cementation and a higher difference in A2-B2 and A1-B1 post-cementation. Among the subgroups, A2 and B2 have a higher MF than A1 and A2, respectively, in both pre- and post-cementation, but with no significant difference in pre-cementation, while in post-cementation in A1-A2, it is practically significant, and the result of B1-B2 is also significant.

## 4. Discussion

The most popular technique for determining how accurately crown restorations fit is the vertical marginal gap measurement [51]. The “perfect margin” means two adjacent surfaces (cement ceramic, cement tooth) that blend into one another without any variation in level and have a continuous margin [52]. The finishing line design (chamfer) and occlusal surface reduction schemes (planar or flat) are advised for zirconia-based restorations, as per manufacturer recommendations.

The planar occlusal reduction scheme of tooth samples is recommended because it entails removing less tooth structure (conservative preparation) under clinical circumstances, thus increasing structural durability and lowering lower risk of dental pulp exposure or injury [53]. In clinical situations involving deep bite, attrition, and insufficient space, which are indicated for full contour zirconia material due to its superior mechanical properties compared with other dental ceramics, zirconia is a type of ceramic material that is known for its strength and durability. It is highly resistant to wear and fracture, making it an excellent choice for restorations in areas of the mouth that are subject to high stress, such as the molars. Additionally, zirconia is biocompatible, which means that it is unlikely to cause an adverse reaction in the body. Zirconia is also highly aesthetic and can be matched to the color of natural teeth [54]. Tooth samples with overly simplified shapes and a flat occlusal reduction scheme may be necessary. This was consistent with the findings that showed a satisfactory marginal fit was achieved by the flat occlusal scheme [55]. Since the digital microscope is regarded as a direct and non-destructive technology that does not disrupt specimens, it was utilized to measure the marginal gap. Additionally, this technique can be used in clinical settings and is the most popular way to measure the vertical gap [56]. The magnification of 230× used in this study was large enough to view the vertical marginal discrepancies accurately. Holden et al. (2009) utilized four distinct points at four locations to assess marginal gap [46]. In this current study, the luting agent was resin-modified GIC-Fuji Plus compared with resin cement from Tokuyama (EsteCem^®^ II Plus, Metelen, Germany). EsteCem^®^ II Plus is a dual-cure resin cement that simplifies indirect restorations. It does not require additional primers or activators and is compatible with all restorative materials. For long-lasting and aesthetically pleasing results, the key characteristics are minimum water absorption, easy cleanup, and exceptional bond strength (Tokuyama Dental America Inc., Tokyo, Japan). Regarding resin-modified glass ionomer cement, due to the chelating between the carboxyl groups of the cement and the calcium and phosphorus of the dentin and enamel apatite, the ionomer cement sticks to the tooth surface after forming ionic bonds. Nonetheless, because of their composite component, RMGICs have a stronger bond with dentine [57].

The outcomes of this in vitro study revealed statistically significant differences for each of the investigated groupings; however, they were all still within the clinically acceptable limit (120 μm). Only in subgroup B2 was the marginal gap 134.806 after cementation. This study’s mean marginal gap values were lower for teeth generated with both types of cement and a chamfer finishing line utilizing a planar occlusal reduction approach than for teeth made using a flat occlusal reduction technique, according to a statistical analysis of this study’s data. This could result from the occlusal–axial line angle of the flat occlusal reduction scheme being at a straight angle, which could impede the proper seating of the crown repair. A planar occlusal reduction approach and chamfer finishing line indicate lower mean marginal gap values. This could be due to the chamfer finishing line design, which has a more rounded angle between the gingival and axial seats to allow for a more precise seat for crown repair.

During the setting of the crown, luting agents undergo dimensional changes, being soluble in the fluid of the oral cavity and having distinct viscosity and film thickness, affecting the marginal gap. A comparison of the pre-cementation results with those recorded post-cementations for all examined subgroups demonstrated that the luting cement and cementation process have a significant impact on the ultimate precision of the marginal fit for all ceramic crown restorations. The viscosity of the resin cement increases too quickly to flow toward the cervical area and is pushed outside the boundaries of the crown [58]. As a result, more cement is released and pressure is applied, pushing the cement upward, which leads to a significant buildup of luting cement on the prepared tooth’s occlusal surface and may prevent the crown restoration from properly seating post-cementation [59]. Several studies concluded that marginal gap values for all ceramic crown restorations were significantly higher after cementation but within the acceptable clinical limit (120 μm) [60,61].

In this current study, microleakage was seen with all types of cement; however, resin-modified glass ionomer cement had minimum microleakage compared with resin cement. It was reported that results consistent with the impact of cement type, viscosity, and the cementation process could cause this. The adhesion of resin-modified glass ionomer cement is affected by the molecular interactions between the glass ionomer particles and the tooth substance. Using various ceramic materials with varying degrees of precision is just one factor in producing a better seal; other factors include cement solubility and film thickness [62].

We suggest the following:

Study the effects of different finish line designs on marginal fit.

Study the compressive strength of monolithic zirconia crowns with modified vertical preparation using different cement spacers.

Evaluate the impact of various luting agents and cement space geometry set in CAD-CAM on the fit of monolithic zirconia crowns.

Evaluate and compare the effect of different types of luting agents (Rely XTM Ultimate adhesive luting agent, Rely XTM Unicem 200 self-adhesive luting agent, and Riva luting plus glass ionomer) on the internal fitness of monolithic zirconia crowns.

Compare the effect of the digital impression technique with the conventional impression technique on the marginal fit of a monolithic zirconia crown.

This study has the following limitations:

This study has two potential limitations including sample size and time constraints.

The sample size (40 extracted teeth) was too small to identify the significant impact of different occlusal reduction schemes and cements on the marginal adaptation of zirconia crowns.

The time available for this study (3–6 months) was not enough to measure other parameters like the internal adaptation of crowns and the micro-hardness of cement.

## 5. Conclusions

This in vitro investigation showed that all groups’ mean values for the marginal gap of zirconia crowns were within the clinically acceptable range. However, the different occlusal reduction schemes (planar and flat) exhibited statistical differences in terms of marginal accuracy. The planar occlusal reduction scheme with Fuji plus cement showed the lowest mean marginal gap. In contrast, the flat occlusal reduction scheme with Duo Estecem II cement showed the highest marginal gap after cementation. In conclusion, both occlusal reduction schemes (planar and flat) exhibit statistical differences in terms of marginal accuracy, and either of them can be used to fabricate zirconia crown restorations.

## Figures and Tables

**Table 1:** The Shapiro–Wilk and Kolmogorov–Smirnov tests were also used to determine the distribution of the collected data (pre-cementation).

Tests of Normality							
Tested Groups	Subgroups	Kolmogorov–Smirnov			Shapiro–Wilk		
		Statistic	df	Sig.	Statistic	df	Sig.
**A**	**AI**	0.180	10	0.21	0.918	10	0.339
	**A2**	0.174	10	0.23	0.926	10	0.407
**B**	**B1**	0.228	10	0.151	0.846	10	0.052
	**B2**	0.221	10	0.184	0.886	10	0.153

**Table 2:** Descriptive and statistical test of the vertical marginal gaps for the four different subgroups measured in micrometers.

Groups	Subgroups	N	Pre-Cementation			
			Min.	Max.	Mean	±SD
**A**	**A1**	10	47.15	111.25	80.284	23.021
	**A2**	10	68.35	110.85	95.372	13.407
**B**	**B1**	10	66.37	117.32	101.21	14.278
	**B2**	10	104.52	136.67	118.597	9.956

**Table 3:** Student’s *t*-test for the comparison of marginal gaps between each different pair of subgroups.

Paired Samples Test									
Tested Groups/PRE		Paired Differences					*t*	df	Sig. (2-Tailed)
		Mean	Std. Deviation	Std. Error Mean	95% Confidence Interval of the Difference				
					Lower	Upper			
Pair 1	A1-A2	−4.03300	29.42142	9.30387	−25.07981	17.01381	−0.433	9	0.675
Pair 2	A1-B1	−20.96700	32.56010	10.29641	−44.25909	2.32509	−2.036	9	0.072
Pair 3	A1-B2	−23.76000	22.58261	7.14125	−39.91463	−7.60537-	−3.327	9	0.009 **
Pair 4	A2-B1	−16.93400	17.58250	5.56007	−29.51176	−4.35624-	−3.046	9	0.01 *
Pair 5	A2-B2	−19.72700	15.64231	4.94653	−30.91684	−8.53716-	−3.988	9	0.003 **
Pair 6	B1-B2	−2.79300	17.11347	5.41175	−15.03524	9.44924	−0.516	9	0.618

* highly significant. ** very highly significant.

**Table 4:** ANOVA-post hoc/LSD multiple comparisons.

Statistics/PRE	AI	A2	B1	B2
N	10	10	10	10
Mean	A 80.284	B 95.372	C 101.21	D 118.597
±Std. Deviation	23.02	13.407	14.278	9.959
LSD	3.012			
*p* value	F = 6.22 *p* value = 0.002 H.SIG			

The LSD test was used to calculate the significant differences between the tested mean, and the letters (A, B, C, and D) represent the levels of significance, with the letter (A) representing the highest level and letter (D) representing the lowest. *p* < 0.05 was considered statically significant. The red mean highly significant.

**Table 5:** The Shapiro–Wilk and Kolmogorov–Smirnov tests were also used to determine the distribution of the collected data.

Tests of Normality						
Tested Groups Post	Kolmogorov–Smirnov			Shapiro–Wilk		
	Statistic	df	Sig.	Statistic	df	Sig.
A1	0.146	10	0.21	0.937	10	0.515
A2	0.165	10	0.23	0.922	10	0.375
B1	0.145	10	0.12	0.932	10	0.466
B2	0.190	10	0.22	0.943	10	0.592

**Table 6:** Descriptive statistics of the vertical marginal gaps for the four different subgroups measured in micrometers.

Groups	Subgroups	N	Descriptive Statistics/Post-Cementation			
			Min.	Max.	Mean	±SD
A	A1	10	73.07	122.05	95.442	17.598
	A2	10	91.45	119.30	108.424	8.807
B	B1	10	82.82	137.75	116.758	16.151
	B2	10	119.60	146.55	134.806	8.114

**Table 7:** Student’s *t*-test for the comparison of marginal gaps between each different pair of subgroups.

Paired Samples Test									
Tested Groups/Post		Paired Differences					*t*	df	Sig. (2-Tailed)
		Mean	Std. Deviation	Std. Error Mean	95% Confidence Interval of the Difference				
					Lower	Upper			
Pair 1	A1-A2	−12.98800	20.69533	6.54444	−27.79255	1.81655	−1.985	9	0.078
Pair 2	A1-B1	−21.23300	29.35672	9.28341	−42.23353	−0.23247	−2.287	9	0.0481 *
Pair 3	A1-B2	−39.36400	18.20305	5.75631	−52.38568	−26.34232	−6.838	9	0.0001 ***
Pair 4	A2-B1	−8.24500	21.88527	6.92073	−23.90078	7.41078	−1.191	9	0.264
Pair 5	A2-B2	−26.37600	12.10464	3.82782	−35.03514	−17.71686	−6.891	9	0.0001 ***
Pair 6	B1-B2	−18.13100	20.75647	6.56377	−32.97928	−3.28272	−2.762	9	0.022 **

* significant.** highly significant. *** very highly significant.

**Table 8:** ANOVA-post hoc/LSD multiple comparisons.

Statistics/Post	A1	A2	B1	B2
N	10	10	10	10
Mean	A 95.442	B 108.424	B 116.758	C 134.806
±Std. Deviation	17.598	8.807	16.151	8.114
LSD	5.38			
*p* value	F = 15.09 *p* value = 0.0001 H.SIG			

The LSD test was used to calculate the significant differences between the tested mean, and the letters (A, B, and C) represent the levels of significance, with the letter (A) representing the highest level and letter (C) representing the lowest. The same letters mean there are no significant differences between the tested mean. *p* < 0.05 was considered statically significant. The red mean highly significant.

**Table 9:** Results of comparative statistical analysis of all tested sub-groups involved in the study pre- and post-cementation.

Statistics	A1	A2	B1	B2
N	10	10	10	10
pre Mean ± SD	80.28 ± 23.02	95.37 ± 13.41	101.21 ± 14.28	118.59 ± 9.95
Post Mean ± SD	95.4 ± 17.6	108.4 ± 8.81	116.76 ± 16.2	134.81 ± 8.1
*t* test	2.74	29.5	5.08	75.5
*p* value	0.115	0.000 *	0.037 *	0.000 *

* highly significant.

## Data Availability

Data are contained within the article.
